# Disseminated cryptococcosis in a patient with idiopathic CD4 + T lymphocytopenia presenting as prostate and adrenal nodules: diagnosis from pathology and mNGS, a case report

**DOI:** 10.1186/s12879-023-08926-1

**Published:** 2024-01-02

**Authors:** Liu Baomo, Zeng Guofen, Dong Jie, Xie Liu, Chen Shuru, Liu Jing

**Affiliations:** 1 Department of Pulmonary and Critical Care Medicine, The First Affiliated Hospital of Sun Yat-sen University, Guangzhou, China; 2 Department of Infectious Diseases, Third Affiliated Hospital of Sun Yat-sen University, 510630 Guangzhou, China; 3 Department of Infectious Diseases, Zhuhai People’s Hospital(Zhuhai Hospital affiliated with Jinan University), 519000 Zhuhai, China; 4 Department of Infectious Diseases, Shenzhen Longhua District People’s Hospital, 518110 Shenzhen, China

**Keywords:** Disseminated cryptococcosis, Idiopathic CD4 + T lymphocytopenia, Prostate, Adrenal grand Infection, mNGS of FFPE.

## Abstract

Disseminated Cryptococcosis infection typically occurs in immunocompromised patients, often manifested as pneumonia or meningoencephalitis. Cases with involvement of either prostate or adrenal glands are less frequent. We describe a case of an immunocompromised 62-year-old man with new-found Idiopathic CD4 + T lymphocytopenia who presented with urinary irritation symptoms followed by headache. The patient was finally diagnosed as disseminated cryptococcosis of prostate, adrenal gland involvement with the help of combining histopathology of formalin-fixed, paraffin-embedded tissue with metagenomic next-generation sequencing technique to identify *C neoformans sensu stricto* in prostate, adrenal gland tissues. Clinicians should be aware of atypical presentations of cryptococcal disease. In this case of cryptococcosis in immunocompromised patients, we find that cryptococcosis can affect varied organs simultaneously and should be considered in the differential of infectious diseases. And mNGS technology helps to confirm the diagnosis.

**Supplementary Information:**

The online version contains supplementary material available at 10.1186/s12879-023-08926-1.

## Background

Disseminated cryptococcosis is a widely distributed invasive fungal infection disease caused by *Cryptococcus* species that poses substantial therapeutic challenges. Cryptococcosis has been attributed to two fungal species, *Cryptococcus neoformans* and *Cryptococcus gattii*. In China, *C neoformans sensu stricto* is particularly abundant in the environment and a major causal agent for most of cryptococcal infections [1]. The most of patients with disseminated cryptococcosis are apparently immunocompromised, such as with solid organ transplantation, corticosteroid use or malignancy. Idiopathic CD4 + T lymphocytopenia (ICL) is a rare syndrome with an unexplained deficiency of CD4 + T lymphocytes in circulating blood and no sign of Human Immunodefciency Virus (HIV) infection. *Cryptococcus* infection is the most frequent opportunistic infection among ICL patients with 26.6% ICL patients(total 258 cases) being reported to have cryptococcosis [2].

The lungs and central nervous system (CNS) are the organs frequently affected by disseminated cryptococcosis. Involvements of other organs, including thyroid, liver, kidneys, pancreas, lymph nodes, spleen, intestine, and ovaries by *Cryptococcus* are also sporadically reported [3]. To the best of our knowledge, co-involvement of prostate and adrenal glands in cryptococcosis caused by *C neoformans sensu stricto* has been scarcely mentioned in human. Especially past studies generally believed that cryptococcosis started with inhalation of fungal cells from the bird droppings accumulated environment. Immunocompromised patients may develop pneumonia in the lung from *Cryptococcus* species, which could then spread to other organs by blood invasion [4]. Cryptococcosis patients with urinary irritation symptoms at disease onset are uncommon.

Early and quick identification of fungi are essential for the timely initiation of suitable antifungal therapy in patients with cryptococcosis. The diagnosis of disseminated cryptococcosis typically relies on antigen testing of serum or cerebrospinal fluid, pathogens culture or histopathology of infected tissues [5]. However, these mentioned methods not only need longer time to confirm but also cannot identify the fungi species. The recognition of fungi species is critical to select appropriate antifungal therapy. Recent studies have reported that the molecular identification of fungi from formalin-fixed and paraffin-embedded (FFPE) tissues with metagenomic Next-generation sequencing(mNGS) could overcome the above disadvantages and detect the fungus to the genus level [6]. The application of mNGS of FFPE samples for assistant diagnosis of cryptococcosis in realistic clinical condition is rarely reported.

Here, we present the case report of disseminated cryptococcosis with involvement of the prostate and adrenal glands in an ICL-related-immunocompromised 62-year-old male, whose diagnosis depending on combination of histopathology and mNGS of FFPE slides, treated at our hospital’s infectious diseases department. After intravenous anti-*Cryptococcus* therapy, the patient achieved obvious clinical remission. Publication of the clinical data was approved by the patient.

## Case presentation

A 62-year-old man had no past medical history of any disease and had quit smoking and drinking for more than 20 years. The patient was a farmer living in the countryside of Zhanjiang, Guangdong, and had not travelled outside, and had no contact with animals or bird droppings.

More than two months prior to admission, he presented with frequent, urgent and painful urination. Then the patient developed severe left-sided headache, left eye pain with tears. Meantime, he had short period of fever with maximum body temperature 37.8 degrees. He denied blurred vision, jet vomiting, limbs weakness, dizziness, tinnitus and other discomfort. The cranial computerized tomography (CT) in local hospital showed multiple and nature-undetermined abnormal intracranial signal foci, and right adrenal gland occupancy (benign). B-ultrasound examination of the urinary system showed that the prostate was enlarged, with a size of 38 × 47 × 36 mm. After symptomatic support treatment, the patient’s symptoms could be relieved.

**Fig. 1:**
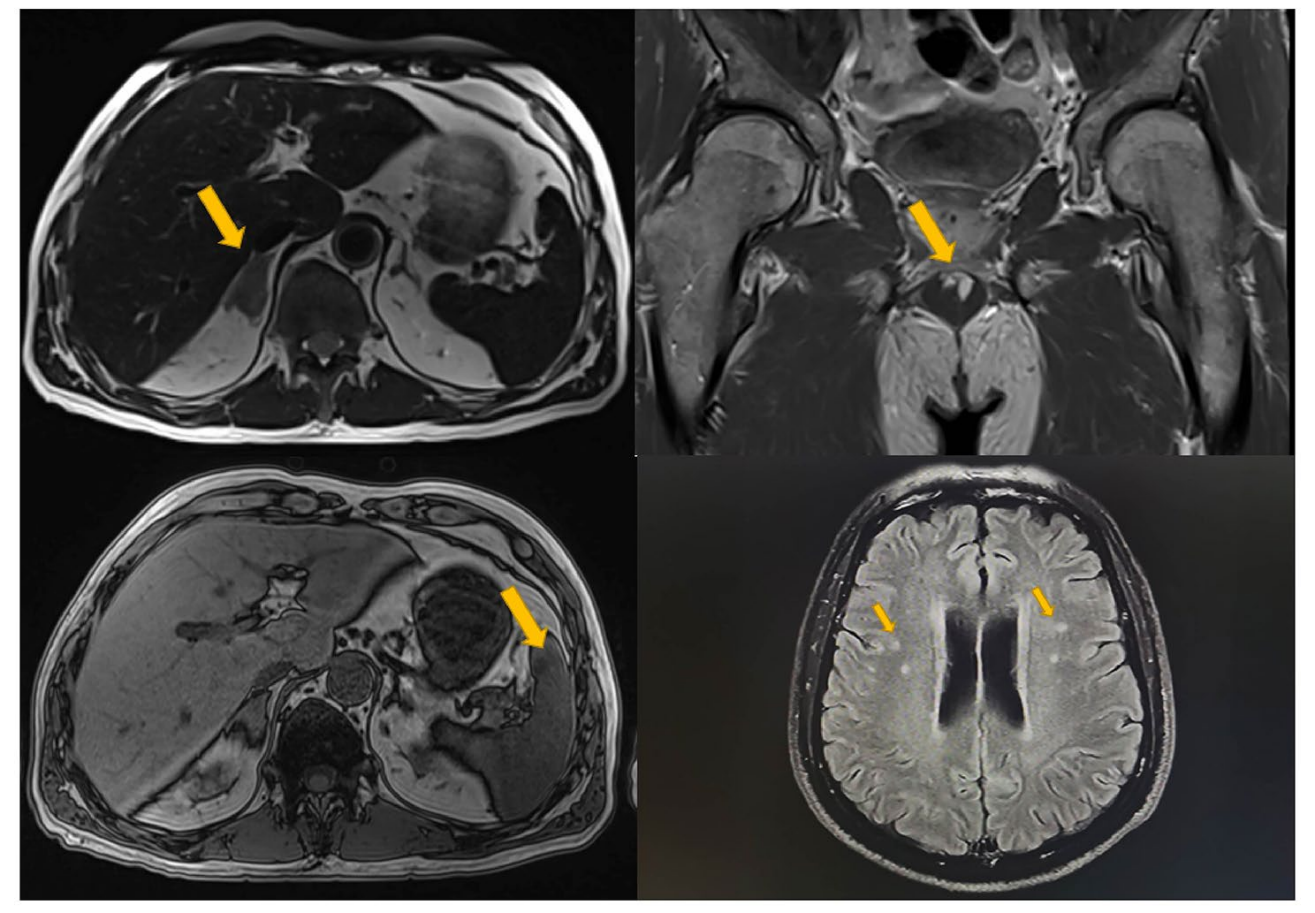
Multiple nodules found in MRI(upper left: adrenal gland, left bottom: spleen, upper right: prostate, right bottom: brain.)

The patient was suspected of having prostate cancer with brain and systemic metastases. More than a month prior to admission, the patient attended the urology department of the second hospital for further diagnostic. Prostate-specific membrane antigen (PSMA) positron emission tomography (PET) -CT demonstrated prostate with slightly increased PSMA expression, the thickened right adrenal gland with active FDG metabolism and low expression of PSMA, and multiple small nodules in bilateral lungs with inactive FDG metabolism. A magnetic resonance imaging (MRI) of head and abdomen further revealed several nodules were found in the brain, including the right parietal lobe, bilateral frontal lobes, bilateral temporal lobes, left basal ganglia, left thalamus, right hippocampus, inferior pontine and subcortical area. In addition, the spleen also showed many nodules of unknown nature (Fig. 1).

**Fig. 2:**
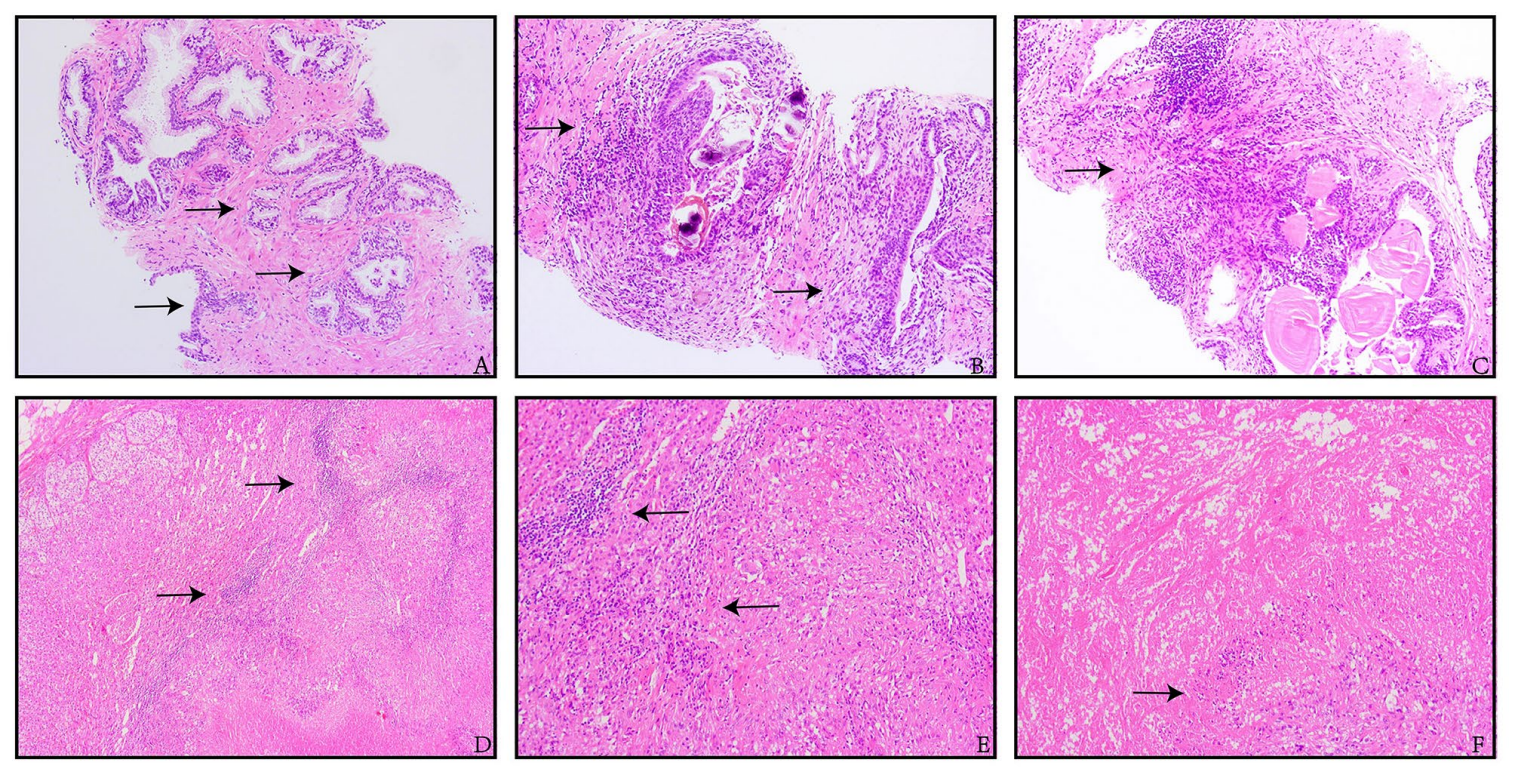
Pathology of prostate and adrenal gland.(**A-C** show prostate tissue and **D-F** show right adrenal gland. The arrows indicate the granulomatous inflammation. Figure 2**A** and **D** were magnified x10. Figure 2**B, C, E, F** were magnified x20.)

Biopsy workup was initiated to identify the nature of multiple nodules in the body. Three weeks before admission, ultrasound-guided prostate puncture was performed and pathological biopsy showed that prostate tissue was hyperplastic, accompanied by granulomatous inflammation and necrosis, and fungi were seen. Immunohistochemistry showed 34BE12(+), P63(+) and rare P504S. Special dyeing of tissue indicated silver hexamine (+) and acid-fast dyeing (-). Two weeks after prostate needle biopsy, the patient underwent right laparoscopic adrenalectomy. Adrenal gland pathology reported adrenal granulomatous inflammation with necrosis, immunohistochemistry showing CD68(+), CK(-), and special staining showing silver hexamine(+), acid-fast staining(-)(Fig. 2). *Cryptococcus* was suspected to be a possible pathogen for prostate and adrenal gland infection.

He further sought for medical assistance in our hospital. On admission, cryptococcus antigen testing (lateral flow assay, LFA method) of patient’s serum was positive. Although head MRI in our hospital still showed multiple nodules found in the brain but less than before, no cryptococcalcells were detected in the cerebrospinal fluid which was also negative for cryptococcal antigen and cryptococcal culture. A lumbar puncture revealed cerebrospinal fluid with slightly high level of protein(0.459 g/L), normal glucose level(3.62mmol/L) and normal neutrophil count. Previous prostate and adrenal tissue biopsy confirmed microorganisms were consistent with fungi, on a background of focal necrotizing and granulomatous inflammation infiltrate, but it does not provide a genus and/or species identification. A mNGS assay named Q-mNGS (this method introduces spike for quantification of pathogen nucleic acids and facilitates the detection of pathogen identification in tissues. See details in reference [7]) was used to detect fungal etiology in formalin-fixed paraffin embedded (FFPE) prostate and adrenal tissue, which identified *C neoformans sensu stricto.* Moreover, during hospitalization, patient’s blood tests suggested that the lymphocyte cells count in peri-blood was low. Further Lymphocyte classification showed that CD4 + T lymphocytes in circulating blood was 98.3/ul with HIV antigen negative.

With the diagnosis of disseminated cryptococcal infection with prostate, adrenal gland involvement (with suspected lung, brain tissue, spleen involved), the patient was treated with induction treatment for two weeks, including amphotericin B deoxycholate 30 mg given intravenously once a day and flucytosine 1 g given orally four times a day. Due to the progressive increase of creatinine after amphotericin B, the treatment was changed to fluconazole 600 mg orally once daily plus flucytosine 1 g orally four times a day. The patient was discharged from the hospital on regular medication and returned to the hospital for follow-up after four months. The patient is in good general condition, and the urinary tract irritation has largely resolved, with occasional mild headache. Fluconazole was used as a maintenance and secondary treatment for a year.

## Discussion and conclusion

Cryptococcosis, caused by encapsulated *Cryptococcus*, is the third most prevalent invasive fungal infection after aspergillosis and candidiasis. Lung and CNS are the most common involved organs followed by cutaneous systems [4]. However, it’s unusual to find pathogens in prostate, and adrenal gland. To our knowledge, two documents from 1954 to 2022 recorded the co-infection of prostate and adrenal gland. The reported case here indicates that clinicians need to recognize this entity more frequently and be more cognizant of *Cryptococcus* infections in varied organs.

Adrenal glands involvement in cryptococcosis is often companied with adrenal insufficiency (AI) among immunocompromised patients [8]. Our case is different from the prior publications because this patient had no adrenal insufficiency occurred and no overt clinical symptoms correlated with AI (supplementary data 1), which may be related to the left adrenal compensation. This case highlights the significance of taking adrenal gland involvement in cryptococcal infections into account, and conversely to consider the probability of *Cryptococcus* infection in patients with adrenal lesions, in order to improve their outcome with adrenalectomy, if necessary.

*C. neoformans* could induce prostatitis in immunocompromised hosts [9, 10]. The prostate may not only act as a focal point for systemic relapse but also continue to be immune to therapy [11]. Genitourinary manipulation may provoke cryptococcal fungemia in immunosuppressed hosts. However, we report a case in which the patient presented as urinary tract irritation at onset and subsequently developed multiple infection lesions throughout the body, which seemed to contradict the viewpoint put forward in the previous study. A few reports believed that the urinary tract might be a portal of cryptococcosis invasion and, urinary symptoms or prostatism might be the initial symptoms in disseminated cryptococcal infection [10, 12, 13]. However, it should be noted that prostatic cryptococcosis is easily misdiagnosed as carcinoma like the patient in this case, resulting in delayed diagnosis or treatment. Early prostatic biopsy could provide precise diagnosis and adequate treatment. Physicians should increase awareness of fungous infection with prostate involved in the immunodeficient population.

*C neoformans sensu stricto* belongs to serotype A of *C. neoformans* based on antigenic differences and has specific distribution in clinical and environmental sources. Compared with *C. neoformans var. neoformans* (serotype D) or *Cryptococcus gattii*, *C neoformans sensu stricto* showed higher virulence in immunocompromised patients and heat-tolerance in condition with high fever [14]. Furthermore, Desnos-Ollivier, M. et al. even observed a worse prognosis for infections caused by serotype A *Cryptococcus* compared with those caused by serotype D or other serotypes [15]. A nationwide molecular epidemiological survey of *Cryptococcus* in China demonstrated that there was a significant increase in proportion of *C neoformans sensu stricto* isolates with higher MICs to fluconazole. But *C neoformans sensu stricto* isolates maintained continuous susceptibility to amphotericin B and 5-flucytosine, which were still recommended as first-line treatment in immunosuppressed patients with cryptococcosis [16]. These drugs reach therapeutic concentrations in prostatic fluid and serum. The patient of this study acquired clinical remission after intravenous and oral medication of above medicines.

An investigation for an underlying immunosuppressing disease is necessary for each patient with cryptococcal infection, as cryptococcosis defined as an opportunistic infection. The patient reported in this case was immunocompromised and complicated with ICL, with the evidence of CD4 + T lymphocytes in circulating blood was lower than 300 cells/ul. And several routine tests of his blood found lymphocyte count was less than 0.6 × 10^9^/L. However, it must be stressed that the strict diagnostic criteria of ICL are absolute CD4 count less than 300 cells/ul on more than 1 occasion [17]. Further follow-up of CD4 counts of this patient is required. Currently, ICL has unclear etiology and treatments of ICL rely on antimicrobial agents for opportunistic infection like cryptococcosis. Other therapeutic options, including interleukin-2 or intravenous immunoglobulin have controversial therapeutic effect [18].

Definitive diagnosis of cryptococcosis is made by histopathology, fungal culture, and serology. Among them, histopathology is the gold standard [19], but it does not identify fungi to a genus and/or species. Moreover, it is time-consuming leading to delayed diagnosis and subsequent treatments. Herein, we employed mNGS technique to identify the etiology of tissues that have been formalin-fixed, paraffin-embedded (FFPE). The patient was initially mistaken as prostate carcinoma, and we missed the opportunity to acquire his fresh tissues for *Cryptococcus* culture. Histopathology of his prostate and adrenal gland found fungi but no evidence of fungal species. Combining formalin-fixed, paraffin-embedded tissue with mNGS technique identified *C neoformans sensu stricto* and offered deterministic diagnosis for this patient. In FFPE tissues, mNGS has been shown to be a potent tool for reliable and unbiased fungal identification [6]. Although fungal DNA degradation in FFPE may affect molecular testing, studies proven that fungal DNA has been successfully recognized in tissue pieces for up to 3 ~ 7 years. Paige M K Larkin et al. reported the organisms identified by mNGS in FFPE showed > 90% concordance with pathogens recognized by the histopathologic morphology and surgical pathologist [20]. Moreover, molecular biology integrated with FFPE tissue has been applied in clinical diagnosis, which helped to change the etiology to a more aggressive fungus and result in appropriate antifungal treatment [21, 22]. We demonstrate that the mNGS method performs excellent on FFPE samples, and it can be applied to pathological diagnosis of disseminated cryptococcosis in clinical condition. However, its high price may limit its use in resource-poor countries.

In 2018, the World Health Organization revised international guidelines to recommend amphotericin B deoxycholate (AmBd) and flucytosine for induction therapy in patients with invasive fungal infections from resource limited settings based on ACTA clinical trial [23]. The infectious diseases society of America updated guideline to suggest that during two weeks of induction treatment, the dosage of AmBd is given by 0.7–1.0 mg per kilogram per day intravenously plus flucytosine (100 mg per kilogram per day orally in 4 divided doses) [24]. However, China experts recommend the dosage of AmBd as 0.5–0.7 mg per kilogram per day considering nephrotoxicity of drug [25]. In this report, the body weight of patient was about 60 kg, so we used AmBd 30 mg per day in two weeks for induction treatment. The formidable nephrotoxicity of AmBd should be noticed, which became a vital clinical problem. During 14 days of treatment with AmBd and flucytosine for this patient, we observed serum creatinine increased slowly but did not exceed the normal standard value (58umol/L at Day2 to 116 umol/L at Day14). Subsequent treatment was changed to consolidation phase with oral fluconazole and 5-flucytosine for 6–12 months.

In conclusion, this is a case that manifested as prostate and adrenal gland co-infection caused by *C neoformans sensu stricto*. To our knowledge, we reported the first case of an ICL patient with *Cryptococcus* in prostate and adrenal gland. We present this case to expand the clinical spectrum of *C neoformans sensu stricto*. infection thus help clinicians recognize that patients present with urinary irritation symptoms followed by headache might also be disseminated cryptococcosis infection, especially for those who are immunocompromised, which always be mistaken as prostate malignant tumor with systemic metastasis. Clinicians should pay attention to atypical presentations of cryptococcal disease. Cryptococcosis can potentially affect multiple organs and should be listed in the differential of infectious diseases. Moreover, the combination of histopathology and mNGS in FFPE tissue could be applied to clinical diagnosis or treatment guidance for cryptococcosis.

### Electronic supplementary material

Below is the link to the electronic supplementary material.

Supplementary Material 1

## Data Availability

Not applicable.

## Abbreviations

ICL: Idiopathic CD4 + T lymphocytopenia
mNGS: metagenomic next-generation sequencing
FFPE: formalin-fixed and paraffin-embedded
