# Supplementary material for: Disseminated cryptococcosis in a patient with idiopathic CD4 + T lymphocytopenia presenting as prostate and adrenal nodules: diagnosis from pathology and mNGS, a case report
Source: BMC Infect Dis. 2024 Jan 2;24:26. doi: 10.1186/s12879-023-08926-1 (PMC10763445; doi:10.1186/s12879-023-08926-1)
Supplement: Supplementary file 1 — Supplementary Material 1 [file 12879_2023_8926_MOESM1_ESM.docx]

Supplementary data 1

Endocrine-related test of this patient.

| Time | cortisol (nmol/L) | ACTH adrenocorticotropic hormone(pg/ml) |
| --- | --- | --- |
| 0:00 | 62.55 | 12 |
| 8:00 | 333.62 | 31 |
| 16:00 | 179.05 | 17 |

Aldosterone (decubitus) 50.7 ng/L; (orthostatic) 37.9 ↓ ng/L;

Methoxyadrenaline 0.10 nmol/L; Methoxynoradrenaline 0.26 nmol/L;
